# Advancing HIV Drug Resistance Technologies and Strategies: Insights from South Africa’s Experience and Future Directions for Resource-Limited Settings

**DOI:** 10.3390/diagnostics13132209

**Published:** 2023-06-29

**Authors:** Kim Steegen, Gert U. van Zyl, Mathilda Claassen, Aabida Khan, Melendhran Pillay, Subitha Govender, Phillip A. Bester, Johanna M. van Straaten, Vibha Kana, Ewaldé Cutler, Monalisa N. Kalimashe, Ramokone L. Lebelo, Mokopi B. H. Moloi, Lucia Hans

**Affiliations:** 1 Department of Molecular Medicine and Haematology, National Health Laboratory Service, Charlotte Maxeke Johannesburg Hospital, Johannesburg 2193, South Africa; lucia.hans@nhls.ac.za; 2 Department of Molecular Medicine and Haematology, Faculty of Health Sciences, University of the Witwatersrand, Johannesburg 2000, South Africa; 3 Wits Diagnostic Innovation Hub, Faculty of Health Sciences, University of the Witwatersrand, Johannesburg 2000, South Africa; 4 Division of Medical Virology, Stellenbosh University, Stellenbosh 7602, South Africa; guvz@sun.ac.za (G.U.v.Z.); mc@sun.ac.za (M.C.); 5 Division of Medical Virology, Stellenbosh National Health Laboratory Service, Tygerberg Hospital, Tygerberg 7505, South Africa; 6 Department of Virology, School of Laboratory Medicine and Medical Sciences, University of KwaZulu-Natal, Durban 4041, South Africamelendhra.pillay@nhls.ac.za (M.P.); 7 Department of Virology, National Health Laboratory Service, Inkosi Albert Luthuli Central Hospital, Durban 4058, South Africa; subitha.govender@nhls.ac.za; 8 Department of Medical Microbiology and Virology, University of the Free State, Bloemfontein 9300, South Africa; besterpa@ufs.ac.za; 9 Department of Medical Microbiology and Virology, National Health Laboratory Service, Universitas Academic Hospital, Bloemfontein 9301, South Africa; johanna.vanstraaten@nhls.ac.za; 10 Centre for HIV and STIs, National Institute for Communicable Diseases, Johannesburg 2192, South Africa; vibhak@nicd.ac.za (V.K.); ewaldec@nicd.ac.za (E.C.); monalisak@nicd.ac.za (M.N.K.); 11 Department of Virological Pathology, Sefako Makgatho Health Sciences University, Pretoria 0204, South Africa; ramokone.lebelo@nhls.ac.za (R.L.L.); mokopi.moloi@nhls.ac.za (M.B.H.M.); 12 Department of Virological Pathology, National Health Laboratory Service, Sefako Makgatho Health Sciences University, Pretoria 0204, South Africa

**Keywords:** HIV drug resistance testing, surveillance, acquired drug resistance, pretreatment drug resistance, next-generation sequencing

## Abstract

Monitoring of HIV drug resistance (HIVDR) remains critical for ensuring countries attain and sustain the global goals for ending HIV as a public health threat by 2030. On an individual patient level, drug resistance results assist in ensuring unnecessary treatment switches are avoided and subsequent regimens are tailored on a case-by-case basis, should resistance be detected. Although there is a disparity in access to HIVDR testing in high-income countries compared to low- and middle-income countries (LMICS), more LMICs have now included HIVDR testing for individual patient management in some groups of patients. In this review, we describe different strategies for surveillance as well as where HIVDR testing can be implemented for individual patient management. In addition, we briefly review available technologies for HIVDR testing in LMICs, including Sanger sequencing, next-generation sequencing, and some point-of-care options. Finally, we describe how South Africa has implemented HIVDR testing in the public sector.

## 1. Introduction

Sustained high levels of community HIV viral load (VL) suppression are critical to achieving the WHO/UNAIDS 95-95-95 target of eliminating AIDS as a public health threat by 2030 [1]. However, the emergence of HIV drug resistance (HIVDR) can compromise the effectiveness of antiretroviral therapy (ART), which may lead to increasing HIV-associated morbidity and mortality [2]. Several factors contribute to the emergence of HIVDR, including, suboptimal ART adherence, poor access to drugs, prolonged virological failure (VF), drug toxicity, and drug-drug interactions.

HIV drug resistance testing is recommended for individual patient management in many resource-rich settings [3,4,5], but this approach is not always feasible in low- and middle-income countries (LMICs). In the 2019 treatment guidelines, WHO recognized that resistance tests for individual patient monitoring could minimize unnecessary treatment switches when there is susceptibility to the anchor drugs [6]. In addition, genotyping testing can inform optimal salvage regimen composition. However, implementation of HIVDR testing at the patient level in LMICs only adds value when VL testing is available on a large scale, resources and laboratory capacity are available, and the country has access to clinical experts and virologists to interpret resistance results [6].

In countries where routine resistance testing is not feasible, it is advised to conduct regular surveys to monitor: pretreatment drug resistance (PDR) in adults initiating or re-initiating first-line ART, PDR in infants newly diagnosed with HIV, acquired drug resistance (ADR) in adults, adolescents, and children with virologic failure (VF) on ART, and HIV-diagnosed pre-exposure prophylaxis (PREP) recipients [7,8].

Here, we review the impact of HIVDR testing on treatment guidelines and individual patient management in LMICs, as well as what technologies are available and required in the near future to address the need for HIVDR testing with the growing number of patients on ART and changing regimens.

## 2. Current Positioning of HIV Drug Resistance Testing

### 2.1. HIVDR Testing for Surveillance Purposes

Global and national ART guidelines rely on the availability of reliable national data, including treatment outcomes and HIVDR prevalence trends. Now that routine VL testing is widely available, programmatic data can often be used to assess the responses to ARV regimens at a population level. However, population-level implementation of HIVDR testing is limited. Therefore, one has to rely on nationally representative surveillance data instead.

#### 2.1.1. Recommended HIVDR Surveillance Strategies

Over the years, the WHO has published a number of surveillance strategies, targeting different populations and providing tools to calculate target sample sizes for each of the methods.

For the assessment of PDR, a cross-sectional clinic-based survey approach is recommended [9]. In the first stage, a minimum of 15 representative clinics should be selected from all clinics that initiate ART in the country. Once the clinics are selected, consecutive eligible patients initiating ART are enrolled until the predetermined sample size has been reached. The survey should not exceed six months, and where possible, a distinction should be made between ART naïve and previously treatment-exposed patients.

The protocol to assess HIVDR in children less than 18 months of age recommends the use of remnant dried blood spots (DBS) from early infant diagnosis (EID) testing over a period of 12 months [10]. In this approach, all laboratories performing EID testing should contribute specimens, with the number of specimens contributed dependent on individual laboratory test volumes.

Three different methods were proposed to assess the acquired HIVDR, which can be applied to adults, adolescents, and children.

Strategy one: For countries with at least 60% VL testing coverage, a nationally representative laboratory-based method is recommended [11]. It targets patients on ART with a VL ≥ 1000 copies/mL by using remnant specimens from routine VL testing. If the country has a unique patient identifier and the treatment history is available, acquired drug resistance can be assessed in the general population and specifically in the group exposed to dolutegravir (DTG)-based ART. The sampling design uses double stratification, with both the VL testing laboratory and the ART regimen (DTG versus non-DTG) as stratifying variables.

Strategy two: For countries with inadequate VL testing coverage (<60%), a nationally representative clinic-based method is suggested [12]. This approach uses a two-stage cluster design. First, ART clinics are randomly sampled for inclusion using proportion-to-size sampling. Second, eligible individuals within the clinic are sampled, with the first sample drawn from people receiving DTG-based ART and the second sample drawn from people receiving non-DTG-based ART. The recommended maximum period for sample collection is three months.

Strategy three: Should both of these approaches not be feasible, WHO has proposed a sentinel laboratory-based method targeting patients on DTG-based ART [13]. This survey uses a single-stage design applied at selected VL testing laboratories that serve as sentinel sites. Countries may choose to implement the survey at one or more VL testing laboratories. If more than one VL testing laboratory is included, the eligible remnant specimens are sampled at each laboratory until the required target sample size is achieved at each laboratory. The maximum period recommended for the survey is three months, with a target sample size of 139 specimens.

#### 2.1.2. Impact of HIVDR Surveys on ART Guidelines

##### Pretreatment Drug Resistance in Adults and Adolescents

Outcomes of PDR surveys provide information to select nationally recommended first-line ART regimens, especially in countries where baseline HIVDR testing is not available. During the initial ART roll-out phase in LMICs (2004–2010), HIVDR levels among patients initiating treatment remained relatively low, but were estimated to increase at a rate of 29% per year in East Africa and 14% per year in southern Africa [14], with levels of PDR reaching approximately 7% globally by 2010 [15]. More recently, most countries reporting nationally representative survey data showed that >10% of individuals who initiated ART had non-nucleoside reverse transcriptase inhibitor (NNRTI) resistance, with up to a 3-fold higher prevalence among patients with prior ART exposure [2,16,17]. This would compromise the success of the previously recommended efavirenz (EFV)-based treatment. These findings and superior virological outcomes in studies [18] and real-life settings [19] led to the recommendation to implement first-line DTG-based ART. Alternatively, one could perform HIVDR testing prior to treatment initiation in countries without access to DTG. After some initial hesitancy over the massive roll-out of DTG, especially in women of childbearing potential [20], DTG has now been implemented widely, with 80% of patients on DTG-based regimens in 2022. The Clinton Health Access Initiative predicts this to increase and begin to stabilize at approximately 88% in 2023, with a slight rise in the use of protease inhibitors (PIs) as some patients begin to experience treatment failure on DTG [21].

##### Pretreatment Drug Resistance in Infants

Although the implementation of PMTCT regimens has greatly reduced the vertical transmission of HIV-1, at least half of the vertical transmissions involved drug-resistant viruses [2,16,17]. Based on these findings, and studies showing superior outcomes of PI regimens compared to nevirapine, in infants, irrespective of resistance [22,23], WHO already recommended in 2013 that countries no longer prescribe NNRTIs, but initiate children younger than 3 years of age on PI-based ART. Now, the use of DTG is now recommended for infants weighing at least 3 kg and 4 months of age [24]. The uptake of ritonavir-boosted lopinavir was initially slow in infants, due to the unavailability of heat-stable and palatable pediatric formulations in many LMICs. Recent data, however, suggest that the use of NNRTIs in young children has dropped from 75% in 2018 to 29% in 2021, while at least 24 LMICs will have adopted the use of DTG-based pediatric regimens by the end of 2021 [21].

##### Acquired Drug Resistance

Surveillance of ADR provides critical information needed to assess the performance of ART programs in achieving VL suppression targets. The pooled analysis of VL suppression in the African region between 2014 and 2020 was 94% in patients receiving NNRTIs [2]. The resistance profiles observed in populations receiving ART inform the selection of optimal ART regimens. Due to the low genetic barrier of many nucleoside reverse transcriptase inhibitors (NRTIs) and NNRTIs, a high prevalence of ADR is expected in patients failing NNRTI-based ART, especially in regions where VL is not readily available for patient monitoring and patients remain on a failing regimen for an extended period of time. Between 2014 and 2020, more than half of patients failing NNRTI-based ART had evidence of efavirenz and nevirapine resistance. In LMICs, NNRTI resistance ranged from 50 to 97% in patients on NNRTI-based ART for 12 months with VF. Similarly, NNRTI resistance ranged from 50 to 95% among patients on NNRTI-based ART for 48 months with VF [2]. In addition, pooled analysis of African data showed that 85% of patients failing NNRTI regimens had NRTI resistance. Despite the high levels of NRTI and NNRTI resistance in this group, resistance profiles are predictable, and an empiric switch to PI-based ART without the need for HIVDR testing has been proven successful [25,26,27]. Moreover, patients with NRTI resistance at the time of the switch from NNRTI to PI-based ART were more likely to reach viral suppression [28]. Substantial residual activity of the NRTI backbone, despite the presence of extensive NRTI resistance, does not support the use of genotypic resistance testing after NNRTI treatment failure [28].

Surveillance data on resistance profiles in patients failing PI-based ART is scarcer and was not included in the WHO resistance reports. However, a systematic review and meta-analysis of studies reporting outcomes of PI-based second-line ART in sub-Saharan Africa showed viral suppression rates of 69% and 58% at 48 and 96 weeks, respectively. Major protease resistance mutations were observed in 17% of those failing second-line PI-based ART. This prevalence, however, increased with the duration of second-line ART [29]. In Namibia, the prevalence of PI resistance was found to be 13% in patients with VF on PI-based second-line ART [30]. A nationally representative survey in South Africa showed that 16% of patients with VF receiving PI-based ART had at least one major PI mutation [31]. A more recent study from South Africa showed 33% PI resistance in patients with VF on PI-based regimens with suspected resistance who were referred for HIVDR testing for individual patient management [32]. Clinician bias toward suspected PI resistance might have contributed to the higher PI resistance prevalence compared to the national survey data. These data suggest that PI resistance is less predictable and not as common as NNRTI resistance. For this reason, many countries have implemented the recommendation to use HIVDR testing for individual patient management in patients failing PI-based ART to inform the selection of third-line ART regimens. Some alternative approaches, including clinical prediction rules [33] and ARV drug level testing [34], are being used as screening tools to identify patients at the highest risk of PI resistance who would benefit most from HIVDR testing.

Given the recent large-scale roll-out of DTG, surveillance data on DTG resistance are still uncommon. Only four countries reported viral suppression rates for patients on DTG-based ART in the latest WHO HIVDR report [2]. Viral suppression reached at least 90% in all countries (numbers from Vietnam and Myanmar were very small). Only Zambia included resistance testing in patients failing DTG-based ART, but no INSTI mutations were detected. As more countries scale up the transition to DTG-based ART, surveillance for DTG-resistant viruses in LMICs is crucial.

### 2.2. HIV Drug Resistance Testing for Individualized Patient Care

In high-income countries where individualized treatment is available, HIVDR testing is recommended at the time of VF [3,4,5]. Although access to HIVDR testing for individual patient management has increased in LMICs [2], the testing remains limited to certain populations, such as patients failing PI-based ART. In several countries, including South Africa, genotypic confirmation of PI resistance is required prior to a switch to a third-line regimen [35]. The prevalence of major PI mutations remains low in most patients with VF on second-line ART [29,30,31,32]. For these reasons, the implementation of HIVDR testing for patients failing PI-based ART is warranted in LMICs, especially since drug choices for third-line regimens are limited and often the last treatment options for these patients.

A recent meta-analysis of studies assessing the efficacy, safety, and tolerability of DTG in first-line ART showed that none of the patients developed DTG resistance, indicating most failures are due to suboptimal treatment adherence [36]. Although we do need to monitor this in routine practice, it is unlikely that resistance testing will be implemented in LMICs for patients failing first-line DTG-based ART in the near future.

Switching patients from a NNRTI-based ART to tenofovir-lamivudine-dolutegravir (TLD) has proven to be a successful strategy, with high levels of viral suppression obtained [37,38,39,40,41,42]. Albeit the small number of patients failing DTG-based ART, the prevalence of DTG resistance in patients with treatment failure was higher than expected: 2/14 (14%) [40], 3/17 (18%) [39], and 8/27 (30%) [43]. Studies in ART-experienced children showed similar results, with 8/36 (22%) [44] and 4/22 (18%) [45] DTG resistance in patients with treatment failure. On the other hand, in some studies, DTG resistance was not detected [37,38] or resistance data was not yet available [42]. Although data is still scarce, it is likely that HIVDR testing for individual patient management would be required for patients with VF on DTG-based second- or third-line regimens and possibly a subset of individuals receiving it as a first-line regimen.

## 3. Technologies for HIV Drug Resistance Testing

Current technologies for HIVDR genotypic testing include Sanger sequencing, next-generation sequencing (NGS), and point mutation assays (PMAs). Although not yet commercially available, efforts are being made to develop point-of-care (POC) HIVDR tests.

### 3.1. Sanger Sequencing

Traditionally, most laboratories use Sanger sequencing to assess resistance in viral populations. This target-specific PCR-based technique produces a single consensus sequence of all virus variants present in at least 15–25% of the viral population in a specimen. When the equipment is accessible, Sanger sequencing is relatively simple to perform, and is relatively inexpensive. Several commercial and laboratory-developed methods are available (Table 1). Commercial assays often provide advantages through workflow simplification and method standardization across laboratories. Sanger sequencing is able to simultaneously interrogate multiple mutations alongside genes of interest, providing comprehensive coverage of the multiple, alternative mutational pathways through which HIV-1 can escape drug pressure. Laboratory personnel without specialized bioinformatics training can easily be trained to analyze the sequence output and generate resistance interpretations for various antiretrovirals by using free resources available online, such as the Stanford HIV Drug Resistance Database (https://hivdb.stanford.edu/ (accessed on 28 June 2023)), Geno2Pheno (https://genafor.org/services.php (accessed on 28 June 2023)), ANRS (https://hivfrenchresistance.org/ (accessed on 28 June 2023)) and Rega (https://rega.kuleuven.be/cev/avd/software (accessed on 28 June 2023)).

### 3.2. Next-Generation Sequencing

Next-generation sequencing (NGS) performs high-throughput, massive parallel sequencing, which enables a high sequence depth for the identification of low-abundance variants. If sufficient sequence coverage is ensured, NGS can detect variants present in 1% of the viral population in a specimen, provided a minimum coverage of 1000 sequences per base is used and the HIV RNA is at least 1000 copies/mL. Below this threshold, it is extremely difficult to distinguish true low-frequency variants from PCR errors [46,47]. The clinical relevance of these low-abundance variants remains unclear, and we need to avoid too early or unnecessary treatment switches, especially in LIMCs where the treatment options are limited.

Apart from increased sequence depth, NGS can be leveraged to increase the coverage across the HIV genome to include protease, reverse transcriptase and integrase, or even perform whole genome sequencing at minimal additional cost. The NGS field is currently dominated by Illumina and Ion Torrent platforms, while other players, such as Oxford Nanopore Technologies, have recently joined the competition. Similar to Sanger sequencing, there are now commercial and in-house methods available to perform HIVDR testing using multiple platforms (Table 1). A variety of free online HIVDR analysis pipelines are available, which can be implemented without the need for extensive bioinformatics training. Global initiatives in response to the SARS-CoV-2 pandemic have made NGS equipment more accessible, including in LMICs. However, some challenges remain with regards to the implementation of NGS for HIVDR testing on a large scale. The cost of the acquisition and maintenance of these specialized instruments remains a hurdle. In many countries, there is no or limited technical support, and importing reagents, which often require reliable cold chain storage, remains difficult and costly. In addition, more specialized technical skills are required to perform these assays, especially where automated solutions are unaffordable.

Although NGS is cheaper than Sanger, when comparing the cost per base, savings can only be realized when multiple samples are pooled in a single run. This practice would make NGS suitable and affordable for surveillance activities where large batches of samples are available for testing. On the other hand, when considering implementing NGS for individual patient management, one has to consider the trade-off between cost and turn-around time. In countries with large HIV epidemics, this could be overcome by centralizing testing.

### 3.3. Point-of-Care Assays

The availability of point-of-care (POC) assays could offer a decentralized testing approach. Most POC assays, though not yet commercially available, are designed on the principle of detecting a handful of clinically relevant mutations rather than providing a comprehensive overview of all mutations present.

Point-of-care, or near POC assays, would reduce the need for laboratory equipment to a minimum while shortening the time to results, with the trade-off of missing the complete HIV genetic sequence information. Recent advances in the development of POC assays for HIVDR testing were reviewed by Chua et al. [48]. One technology, the oligonucleotide ligation-based assay (OLA), which is based on a DNA amplification procedure using three probe-ligated primer sets, is likely the most advanced candidate. The OLA is designed to detect mutations of clinical relevance, allowing visual discrimination between mutant and wild-type variants on a lateral flow device [49]. In addition, mobile software has been developed to simplify and standardize result interpretation [50]. This assay showed good performance in validation and near real-life settings [51,52].

Another interesting technology is the pan-degenerate amplification and adaptation (PANDAA) technology, which is based on quantitative PCR and uses extremely degenerate primers that target specific mutation sites and can cope with high HIV diversity next to the mutation site [53]. This technology has been tested in several settings [54,55] and shows high specificity and sensitivity. However, the need for a quantitative PCR instrument remains a challenge for POC applications.

However, no data has been presented yet on the detection of integrase strand transfer inhibitor mutations for either technology.

## 4. The South African Situation

In South Africa, which has the largest ART program globally, a combination of HIVDR testing strategies is used for individual patient management and surveillance. The National Health Laboratory Service (NHLS) provides a laboratory network covering all patients treated in the public sector, which equates to approximately 80% of the population. In 2013, two laboratories offered HIVDR tests for individual patient management. Three additional laboratories were included in this network from 2015 onward to ensure adequate geographical coverage within the country. Virologists and laboratory personnel from each of the five laboratories established an HIVDR committee with the aim of streamlining technical and quality assurance processes and offering support when technical or logistical challenges are encountered. Since 2013, the South African treatment guidelines have recommended HIVDR testing for patients failing PI-based ART with confirmed VF and at least two years of exposure to PIs [35,56,57,58]. With the large-scale roll-out of DTG in 2019, HIVDR testing is only advised in patients failing second- or third-line DTG-based ART, with confirmed VF and at least two years of exposure to DTG, and requires approval by a clinical expert [56]. In the most recent guidelines, DTG resistance testing is further restricted to patients with >80% adherence and exposure to previous ART regimens. HIVDR testing for first-line DTG-based ART failure is generally not recommended but can be permitted in certain scenarios, such as drug-drug interactions (e.g., DTG not boosted during tuberculosis treatment), upon discussion with an expert [35].

In 2022, the NHLS performed approximately 4500 HIVDR tests for individual patient management, of which most were for patients failing PI-based ART and approximately 5% included integrase testing. When PI or InSTI resistance is identified, a full treatment history is submitted to the Third Line Review Committee via the National Department of Health for consideration. Once consensus is reached, a treatment decision is conveyed to the local facility, and if third-line ART (TLART) is indicated, the drugs are dispatched to the facility on a named patient basis. The South African TLART access program is the first and largest of its kind in the public sector and has over 4000 patients on the database as of August 2022.

The National Institute for Communicable Diseases (NICD) and the Human Sciences Research Council (HSRC) conduct the South African HIV Prevalence, Incidence, Behavior and Communication Household Surveys (SABSSM). The most recent SABSSM survey was conducted in 2017, prior to the DTG rollout. Drug-level testing was used as a proxy for adherence. HIVDR was detected in 27% of patients with VF, but this increased to 75% among those who had discontinued treatment [59]. Laboratory-based surveys using remnant viral load specimens from the NHLS VL testing laboratories were conducted in 2019, 2021, and 2022, and are continuing in 2023. Since treatment information was not available, drug-level testing was used as a proxy for ART exposure. Notably, 41–48% of patients with VL > 1000 copies/mL had undetectable levels of ART, indicating that poor adherence is often the cause of VF. The proportion of patients with detectable NNRTI drug levels decreased from 43% in 2019 to 36% in 2021, but the prevalence of NNRTI resistance remained most common over the years, whereas PI (2.2% in 2019 and 4.1% in 2021) and InSTI resistance (0.2% in 2021, not assessed in 2019) remained very low [60,61].

## 5. Discussion

Addressing and monitoring HIVDR remains an essential component in the fight against HIV/AIDS. Despite the inequality in access to HIVDR testing in different regions of the world, more LMICs now have access to the technologies to perform HIVDR testing for surveillance purposes or individual patient management. In this review, we describe the different methodologies recommended by WHO for HIVDR surveillance and the impact these results have on treatment guidelines. Although these surveillance activities are commendable, they remain a resource-consuming exercise that many countries cannot afford to perform on a regular basis. In addition, the WHO HIV drug resistance report often includes relatively old data due to delays in the sharing of data, data curation, and meta-analysis. As part of the response to the SARS-CoV-2 pandemic, sequencing technologies now have a larger footprint in LMICs. However, reagent costs, investment in continuous maintenance of equipment, and sustained technical skills training remain limited in some regions. Next, we presented a nonexhaustive summary of available technologies for HIVDR testing in LMICs, including Sanger sequencing, next-generation sequencing, and some point-of-care options. We acknowledge that we did not perform a systematic review of the literature, which might have led to the omission of some of the newer technologies under development.

## 6. Conclusions

In order to end the HIV epidemic, we require universal access to treatment and sustained viral suppression at the community level. HIVDR is a major barrier to achieving viral suppression; therefore, the availability of HIVDR testing for individual patient monitoring and adequate surveillance programs are pivotal to ensuring ART efficacy for both individual patients and public healthcare. Continuous investment in the development of newer, more accessible technologies and capacity building in LMICs is required. Skills development for laboratory staff, improved technical support by suppliers, and cost negotiations for reagents should be prioritized.

## Figures and Tables

**Table 1:** Sanger and next-generation sequencing HIV-1 drug resistance assays.

Assay	Gene Coverage	Sequencing Platform	Regulatory Status
HIV-1 Genotyping Kit with Integrase (ThermoFisher Scientific, Waltham, MA, USA)	PR, RT, IN	Sanger	CE-IVD
DeepChek-HIV (Advanced Biological Laboratories, Luxembourg)	PR, RT, IN, gp120	Sanger & NGS	CE-IVD
Sentosa SQ HIV Genotyping (VELA Diagnostics, Singapore)	PR, RT, IN	NGS	CE-IVD
HIV-1 Solution (Arrow Diagnostics, Genova, Italy)	PR, RT, IN, gp120	NGS	CE-IVD
DEEPGEN HIV (Case Western Reserve University, Cleveland, OH, USA)	PR, RT, IN	NGS	Core service
GenoSure PRIme (Monogram Biosciences, South San Francisco, CA, USA)	PR, RT, IN	NGS	Core service
Laboratory developed methods	Various	Sanger & NGS	RUO

PR: protease, RT: reverse transcriptase, IN: integrase, gp120: glycoprotein 120, CE-IVD: European CE marking for in vitro diagnostics, RUO: research use only.

## Data Availability

The data presented in this study are available on request from the corresponding author.

## References

[1] UNAIDS (2015). Understanding Fast-Track: Accelerating Action to End the AIDS Epidemic by 2030.

[2] WHO (2021). HIV Drug Resistance Report 2021.

[3] US DHHS (2023). Guidelines for the Use of Antiretroviral Agents in Adults and Adolescents with HIV.

[4] EACS (2022). European AIDS Clinical Society Guidelines Version 11.1.

[5] Waters L., Winston A., Reeves I., Boffito M., Churchill D., Cromarty B., Dunn D., Fink D., Fidler S., Foster C. (2022). BHIVA guidelines on antiretroviral treatment for adults living with HIV-1 2022. HIV Med..

[6] WHO (2019). Update of Recommendations on First- and Second-Line Antiretroviral Regimens.

[7] WHO (2021). HIV Drug Resistance Stategy: 2021 Update.

[8] Bertagnolio S., Jordan M.R., Giron A., Inzaule S. (2022). Epidemiology of HIV drug resistance in low- and middle-income countries and WHO global strategy to monitor its emergence. Curr. Opin. HIV AIDS.

[9] WHO (2014). Surveillance of HIV Drug Resistance in Adults Initiating Antiretroviral Therapy: Pre-Treatment HIV Drug Resistance.

[10] WHO (2017). Surveillance of HIV Drug Resistance in Children Newly Diagnosed with HIV by Early Infant Diagnosis.

[11] WHO (2021). Laboratory-Based Survey of Acquired HIV Drug Resistance Using Remnant Viral Load Specimens.

[12] WHO (2021). Clinic-Based Survey of Acquired HIV Drug Resistance.

[13] WHO (2023). Sentinel Surveys of Acquired HIV Resistance to Dolutegravir among People Receiving Dolutegravir-Containing Antiretroviral Therapy.

[14] Gupta R.K., Jordan M.R., Sultan B.J., Hill A., Davis D.H., Gregson J., Sawyer A.W., Hamers R.L., Ndembi N., Pillay D. (2012). Global trends in antiretroviral resistance in treatment-naive individuals with HIV after rollout of antiretroviral treatment in resource-limited settings: A global collaborative study and meta-regression analysis. Lancet.

[15] WHO (2012). HIV Drug Resistance Report 2012.

[16] WHO (2017). HIV Drug Resistance Report 2017.

[17] WHO (2019). HIV Drug Resistance Report 2019.

[18] Stellbrink H.J., Reynes J., Lazzarin A., Voronin E., Pulido F., Felizarta F., Almond S., St Clair M., Flack N., Min S. (2013). Dolutegravir in antiretroviral-naive adults with HIV-1: 96-week results from a randomized dose-ranging study. AIDS.

[19] Meireles M.V., Pascom A.R.P., Duarte E.C., McFarland W. (2019). Comparative effectiveness of first-line antiretroviral therapy: Results from a large real-world cohort after the implementation of dolutegravir. AIDS.

[20] Zash R., Makhema J., Shapiro R.L. (2018). Neural-Tube Defects with Dolutegravir Treatment from the Time of Conception. N. Engl. J. Med..

[21] CHAI (2022). 2022 HIV Market Report.

[22] Violari A., Lindsey J.C., Hughes M.D., Mujuru H.A., Barlow-Mosha L., Kamthunzi P., Chi B.H., Cotton M.F., Moultrie H., Khadse S. (2012). Nevirapine versus ritonavir-boosted lopinavir for HIV-infected children. N. Engl. J. Med..

[23] Barlow-Mosha L., Angelidou K., Lindsey J., Archary M., Cotton M., Dittmer S., Fairlie L., Kabugho E., Kamthunzi P., Kinikar A. (2016). Nevirapine-versus Lopinavir/Ritonavir-Based Antiretroviral Therapy in HIV-Infected Infants and Young Children: Long-term Follow-up of the IMPAACT P1060 Randomized Trial. Clin. Infect. Dis..

[24] WHO (2021). Consolidated Guidelines on HIV Prevention, Testing, Treatment, Service Delivery and Monitoring: Recommendations for a Public Health Approach.

[25] Paton N.I., Kityo C., Hoppe A., Reid A., Kambugu A., Lugemwa A., van Oosterhout J.J., Kiconco M., Siika A., Mwebaze R. (2014). Assessment of second-line antiretroviral regimens for HIV therapy in Africa. N. Engl. J. Med..

[26] Hakim J.G., Thompson J., Kityo C., Hoppe A., Kambugu A., van Oosterhout J.J., Lugemwa A., Siika A., Mwebaze R., Mweemba A. (2018). Lopinavir plus nucleoside reverse-transcriptase inhibitors, lopinavir plus raltegravir, or lopinavir monotherapy for second-line treatment of HIV (EARNEST): 144-week follow-up results from a randomised controlled trial. Lancet Infect. Dis..

[27] Group S.-L.S., Boyd M.A., Kumarasamy N., Moore C.L., Nwizu C., Losso M.H., Mohapi L., Martin A., Kerr S., Sohn A.H. (2013). Ritonavir-boosted lopinavir plus nucleoside or nucleotide reverse transcriptase inhibitors versus ritonavir-boosted lopinavir plus raltegravir for treatment of HIV-1 infection in adults with virological failure of a standard first-line ART regimen (SECOND-LINE): A randomised, open-label, non-inferiority study. Lancet.

[28] Paton N.I., Kityo C., Thompson J., Nankya I., Bagenda L., Hoppe A., Hakim J., Kambugu A., van Oosterhout J.J., Kiconco M. (2017). Nucleoside reverse-transcriptase inhibitor cross-resistance and outcomes from second-line antiretroviral therapy in the public health approach: An observational analysis within the randomised, open-label, EARNEST trial. Lancet HIV.

[29] Stockdale A.J., Saunders M.J., Boyd M.A., Bonnett L.J., Johnston V., Wandeler G., Schoffelen A.F., Ciaffi L., Stafford K., Collier A.C. (2018). Effectiveness of Protease Inhibitor/Nucleos(t)ide Reverse Transcriptase Inhibitor-Based Second-line Antiretroviral Therapy for the Treatment of Human Immunodeficiency Virus Type 1 Infection in Sub-Saharan Africa: A Systematic Review and Meta-analysis. Clin. Infect. Dis..

[30] Jordan M.R., Hamunime N., Bikinesi L., Sawadogo S., Agolory S., Shiningavamwe A.N., Negussie T., Fisher-Walker C.L., Raizes E.G., Mutenda N. (2020). High levels of HIV drug resistance among adults failing second-line antiretroviral therapy in Namibia. Medicine.

[31] Steegen K., Bronze M., Papathanasopoulos M.A., van Zyl G., Goedhals D., Van Vuuren C., Macleod W., Sanne I., Stevens W.S., Carmona S.C. (2016). Prevalence of Antiretroviral Drug Resistance in Patients Who Are Not Responding to Protease Inhibitor-Based Treatment: Results from the First National Survey in South Africa. J. Infect. Dis..

[32] Chimukangara B., Lessells R.J., Sartorius B., Gounder L., Manyana S., Pillay M., Singh L., Giandhari J., Govender K., Samuel R. (2022). HIV-1 drug resistance in adults and adolescents on protease inhibitor-based antiretroviral therapy in KwaZulu-Natal Province, South Africa. J. Glob. Antimicrob. Resist..

[33] Cohen K., Stewart A., Kengne A.P., Leisegang R., Coetsee M., Maharaj S., Dunn L., Hislop M., van Zyl G., Meintjes G. (2019). A Clinical Prediction Rule for Protease Inhibitor Resistance in Patients Failing Second-Line Antiretroviral Therapy. J. Acquir. Immune Defic. Syndr..

[34] Hermans L.E., Steegen K., Ter Heine R., Schuurman R., Tempelman H.A., Moraba R., van Maarseveen E., Nijhuis M., Pillay T., Legg-E’Silva D. (2020). Drug level testing as a strategy to determine eligibility for drug resistance testing after failure of ART: A retrospective analysis of South African adult patients on second-line ART. J. Int. AIDS Soc..

[35] NDoH (2023). 2023 ART Clinical Guidelines for the Management of HIV in Adults, Pregnancy and Breastfeeding, Adolescents, Children, Infants and Neonates.

[36] Kanters S., Vitoria M., Zoratti M., Doherty M., Penazzato M., Rangaraj A., Ford N., Thorlund K., Anis P.A.H., Karim M.E. (2020). Comparative efficacy, tolerability and safety of dolutegravir and efavirenz 400mg among antiretroviral therapies for first-line HIV treatment: A systematic literature review and network meta-analysis. EClinicalMedicine.

[37] Keene C.M., Cassidy T., Zhao Y., Griesel R., Jackson A., Sayed K., Omar Z., Hill A., Ngwenya O., Van Zyl G. (2023). Recycling Tenofovir in Second-line Antiretroviral Treatment with Dolutegravir: Outcomes and Viral Load Trajectories to 72 weeks. J. Acquir. Immune Defic. Syndr..

[38] Keene C.M., Griesel R., Zhao Y., Gcwabe Z., Sayed K., Hill A., Cassidy T., Ngwenya O., Jackson A., van Zyl G. (2021). Virologic efficacy of tenofovir, lamivudine and dolutegravir as second-line antiretroviral therapy in adults failing a tenofovir-based first-line regimen. AIDS.

[39] Paton N.I., Musaazi J., Kityo C., Walimbwa S., Hoppe A., Balyegisawa A., Asienzo J., Kaimal A., Mirembe G., Lugemwa A. (2022). Efficacy and safety of dolutegravir or darunavir in combination with lamivudine plus either zidovudine or tenofovir for second-line treatment of HIV infection (NADIA): Week 96 results from a prospective, multicentre, open-label, factorial, randomised, non-inferiority trial. Lancet HIV.

[40] Schramm B., Temfack E., Descamps D., Nicholas S., Peytavin G., Bitilinyu-Bangoh J.E., Storto A., Le M.P., Abdi B., Ousley J. (2022). Viral suppression and HIV-1 drug resistance 1 year after pragmatic transitioning to dolutegravir first-line therapy in Malawi: A prospective cohort study. Lancet HIV.

[41] Paton N.I., Musaazi J., Kityo C., Walimbwa S., Hoppe A., Balyegisawa A., Kaimal A., Mirembe G., Tukamushabe P., Ategeka G. (2021). Dolutegravir or Darunavir in Combination with Zidovudine or Tenofovir to Treat HIV. N. Engl. J. Med..

[42] Mulenga L.F.S., Mweemba A., Fwoloshi S., Mweemba A., Siwingwa M., Sivile S., Kampamba D., Engamba D.C., Mbewe N., Phiri H. Dolutegravir with Recycled NRTIs is Non-Inferior to PI-Based ART: VISEND Trial. Proceedings of the 29th Conference on Retroviruses and Opportunistic Infections.

[43] Van Oosterhout J.J., Chipungu C., Nkhoma L., Kanise H., Hosseinipour M.C., Sagno J.B., Simon K., Cox C., Hoffman R., Steegen K. (2022). Dolutegravir Resistance in Malawi’s National HIV Treatment Program. Open Forum Infect. Dis..

[44] Vavro C., Ruel T., Wiznia A., Montanez N., Nangle K., Horton J., Buchanan A.M., Stewart E.L., Palumbo P. (2022). Emergence of Resistance in HIV-1 Integrase with Dolutegravir Treatment in a Pediatric Population from the IMPAACT P1093 Study. Antimicrob. Agents Chemother..

[45] Turkova A., White E., Mujuru H.A., Kekitiinwa A.R., Kityo C.M., Violari A., Lugemwa A., Cressey T.R., Musoke P., Variava E. (2021). Dolutegravir as First- or Second-Line Treatment for HIV-1 Infection in Children. N. Engl. J. Med..

[46] Ji H., Enns E., Brumme C.J., Parkin N., Howison M., Lee E.R., Capina R., Marinier E., Avila-Rios S., Sandstrom P. (2018). Bioinformatic data processing pipelines in support of next-generation sequencing-based HIV drug resistance testing: The Winnipeg Consensus. J. Int. AIDS Soc..

[47] Ji H., Sandstrom P., Paredes R., Harrigan P.R., Brumme C.J., Avila Rios S., Noguera-Julian M., Parkin N., Kantor R. (2020). Are We Ready for NGS HIV Drug Resistance Testing?. The Second “Winnipeg Consensus” Symposium. Viruses.

[48] Chua R.J., Capina R., Ji H. (2022). Point-of-Care Tests for HIV Drug Resistance Monitoring: Advances and Potentials. Pathogens.

[49] Panpradist N., Beck I.A., Chung M.H., Kiarie J.N., Frenkel L.M., Lutz B.R. (2016). Simplified Paper Format for Detecting HIV Drug Resistance in Clinical Specimens by Oligonucleotide Ligation. PLoS ONE.

[50] Panpradist N., Beck I.A., Vrana J., Higa N., McIntyre D., Ruth P.S., So I., Kline E.C., Kanthula R., Wong-On-Wing A. (2019). OLA-Simple: A software-guided HIV-1 drug resistance test for low-resource laboratories. EBioMedicine.

[51] Panpradist N., Beck I.A., Ruth P.S., Avila-Rios S., Garcia-Morales C., Soto-Nava M., Tapia-Trejo D., Matias-Florentino M., Paz-Juarez H.E., Del Arenal-Sanchez S. (2020). Near point-of-care, point-mutation test to detect drug resistance in HIV-1: A validation study in a Mexican cohort. AIDS.

[52] Duarte H.A., Beck I.A., Levine M., Kiptinness C., Kingoo J.M., Chohan B., Sakr S.R., Chung M.H., Frenkel L.M. (2018). Implementation of a point mutation assay for HIV drug resistance testing in Kenya. AIDS.

[53] MacLeod I.J., Rowley C.F., Essex M. (2021). PANDAA intentionally violates conventional qPCR design to enable durable, mismatch-agnostic detection of highly polymorphic pathogens. Commun. Biol..

[54] Maruapula D., MacLeod I.J., Moyo S., Musonda R., Seatla K., Molebatsi K., Leteane M., Essex M., Gaseitsiwe S., Rowley C.F. (2020). Use of a mutation-specific genotyping method to assess for HIV-1 drug resistance in antiretroviral-naive HIV-1 Subtype C-infected patients in Botswana. AAS Open Res..

[55] Kouamou V., Manasa J., Katzenstein D., McGregor A.M., Ndhlovu C.E., Makadzange T. (2020). Diagnostic Accuracy of Pan-Degenerate Amplification and Adaptation Assay for HIV-1 Drug Resistance Mutation Analysis in Low- and Middle-Income Countries. J. Clin. Microbiol..

[56] NDoH (2019). 2019 ART Clinical Guidelines for the Management of HIV in Adults, Pregnancy, Adolescents, Children, Infants and Neonates.

[57] NDoH (2015). National Consolidated Guidelines: Mother to Child Transmission of HIV (PMTCT) and the Management of HIV in Children, Adolescents and Adults.

[58] NDoH (2013). South African Guidelines for the Use of Antiretroviral Agents in HIV-1-Infected Adults and Adolescents.

[59] Moyo S., Hunt G., Zuma K., Zungu M., Marinda E., Mabaso M., Kana V., Kalimashe M., Ledwaba J., Naidoo I. (2020). HIV drug resistance profile in South Africa: Findings and implications from the 2017 national HIV household survey. PLoS ONE.

[60] Hunt G., Steegen K., Hans L., Cassim N., Diallo K., Briggs-Hagen M., Ayalew K., Raizes E., Macleod W., Carmona S. High levels of HIV drug resistance in adult patients with unsuppressed viral load, measured through routine viral load programme monitoring in South Africa, 2019. Proceedings of the 23rd International AIDS Conference.

[61] Steegen K., Hunt G., MacLeod W., Hans L., Kana V., Kalimashe M.N., Zwane H., van der Walt C., Cutler E., Cassim N. HIV Drug Resistance Surveillance Leveraging on Routine ART Programme Monitoring in South Africa. Proceedings of the INTEREST.

